# Development of Novel Adhesive Bilayer Lyophilized Wafer of Moxifloxacin as a Modern Wound Dressing

**DOI:** 10.22037/ijpr.2021.112962.14081

**Published:** 2021

**Authors:** Hossein Jafari, Vahid Ramezani, Mohsen Nabi-Meibodi, Ali Mohammad Ranjbar

**Affiliations:** a *Department of Food and Drug Control, Faculty of Pharmacy, Tehran University of Medical Sciences, Tehran, Iran. *; b *Department of Pharmaceutics, Faculty of Pharmacy, Shahid Sadoughi University of Medical Sciences, Yazd, Iran. *; c *Department of Pharmacognosy, Faculty of Pharmacy, Shahid Sadoughi University of Medical Sciences, Yazd, Iran.*

**Keywords:** Moxifloxacin, Wound healing, Wafer, Bioadhesive force, Drug release

## Abstract

Wound healing is a complex process and is influenced by different factors. Aimed to enhance the wound healing procedure, the Moxifloxacin bilayer wafer was designed, optimized and evaluated as an advanced wound healing dressing. The wafers were prepared by the lyophilization and casting method. Optimization was done according to the results of bioadhesion force, swelling index, release rate, T_40_ and T_90_ (the time to reach 40% and 90% of release). The optimized wafer was evaluated against *in-vitro *and *in-vivo *efficacy using the disc diffusion method and histologic evaluation after application on the wound. The optimized formulation contained HPMC, MC, gelatin and PVP with mounts of 50 mg, 25 mg, 2 mg and 10 mg respectively. The hydrophilic bilayer wafer is adhered to the wound up to the end of wound healing. Application of optimized formulation led to the healing of wound 6 days faster without any sign of infection. The application of this wafer promoted wound healing and epithelium regeneration without any inflammation.

## Introduction

The wound is a break in the epithelial integrity of the skin. Any trauma, burning, contusion, hematoma, laceration, or abrasion can cause a wound. After a wound happens, repairing skin integrity plays a crucial role in the wound healing procedure.

The wound healing process is categorized into four phases: Hemostasis, Inflammation, Proliferation and Maturation (1). According to George Winter, the moist condition enhance wound healing in comparison with the dry environment (2, 3). The modern wound dress-ings have some advanced properties such as; ability to absorb exudate, providing optimum moisture balance at the wound surface and prevention of maceration of circumambient tissue designed to help wound healing. By losing skin as a defense barrier against the pathogens, the risk of aerobic or anaerobic infections rises due to the existence of high contamination of bacteria in exudate. dressings with high fluid retention levels can absorb the exudates which prevent wound infection (4).

Wafers are one of the modern wound dres-sings with a porous structure which enhances gaseous exchange and water evaporation. Therefore, fluid accumulation and infection risk will decrease (5). In addition, this kind of dressing can be served as a drug delivery system to the wound and are suitable for the delivery of antibiotics and wound healing agents (6).

Moxifloxacin is an 8-methoxy-fluoroq-uinolone with a broad spectrum against gram-positive cocci and atypical pathogens in comparison with the former generation of fluoroquinolone-like ciprofloxacin (7). Also, it can reduce the wound Gram-positive and Gram-negative infections and accelerates the wound healing process. 

In this investigation, aimed to accelerate the wound healing process, we have developed and optimized a bilayer wafer containing Moxifloxacin and evaluate it *in-vitro *and *in-vivo*.

## Experimental

*Materials*

Methyl cellulose (MC, MW: 658.73 g/mole), Gelatin (MW: 180.16 g/mole), Polyvinyl pyrrolidone (PVP, MW: 112.89 g/mole), Propylene glycol (PG) and HPMC (K4M) were purchased from Sigma (USA). Also, Moxifloxacin was purchased from DarouPakhsh Pharmaceutical Co (Iran). All reagents were of analytical grade.

*Experimental Design*

A Box-Behnken method was used to design the experiments in terms of 29 runs. Dependent and independent variables are summarized in Table 1 and the range of independent variables are shown in Table 2. Based on screening outcomes, 50 mg of hydroxypropyl methylcellulose (HPMC) was selected as the main film-forming polymer. The amount of Moxifloxacin (MOX) was used for each formulation was 10 mg. Data were analyzed with multiple regression and variables modeling based on the ANOVA test and *p*-value < 0.05.

*Preparation of formulations*

A different mixture of formulations was prepared according to Table 3. Firstly, different concentrations of HPMC, MC, PVP, gelatin, and propylene glycol (plasticizer) were prepared in an aqueous environment separately. Then, the proper amount of each polymer, Moxifloxacin and water were added to the final dish. The final mixture was mixed until a homogenized mixture appeared. The final mixture was degassed spontaneously after 24 h.

*Preparation of wafers*

A modified drug delivery system was developed by design the bilayer wafers. Films were prepared by the film casting method described by Arahman N (11). After 24 h of drying, the casted layer was ready for the addition of the second layer. By pouring the formulations on the casted layer, the freeze-drying procedure started immediately. Christ freeze dryer instrument (Germany) was used to perform the freeze-drying procedure. Freeze-drying cycles was consist of the freezing phase, main drying phase and final drying. In the main dying phase, the temperature was kept at -56 ºC and the pressure was kept at 0.021 mbar. In the main drying phase, wafers lose most of their water content. To increase the wafer’s stability, after 24 h of the main drying phase, the final drying phase began. At the final phase drying which lasted for 24 h, the temperature was kept at 0 ºC and pressure was fixed at 6.1 mbar.

*Swelling study*

The swelling index of the wafers was analyzed to determine the maximum hydration ability of wafers in contact with simulated wound fluid (SWF) (12). SWF consists of 0.02M calcium chloride, 0.4 M sodium chloride, and 0.08M tris-methylamine in deionized water, and the pH was adjusted at 7.4. Samples were weighted every 15 minutes after immersion in the SWF. The swelling ratio of each wafer was calculated according to below equation:

Swelling index = ((W_t _- W_0_) ÷ W_0_) × 100

As W_t _is the weight of the wafer at different time intervals and W_0 _is the initial weight of the wafer before the hydration. Wafer swelling study lasted for 2 h.

*Bioadhesive strength of wafers*

**Figure 1:**
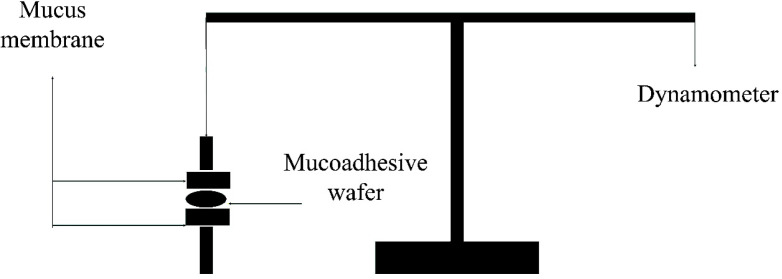
A schematic view of a modified physical balance instrument used in bioadhesion study. Detachment force of rat skin was recorded and reported in the scale of N/Cm^2^

A modified physical balanced instrument described by Gupta et al. (8) was employed to evaluate the bioadhesive strength of wafers (Figure 1). In brief; the shaved rat skin was fixed on two parallel surfaces and wafers were placed between the mucosal part of the skin and stayed steady for 1 min. The maximum force required to detach the skins was recorded as the bioadhesive strength in the scale of N/Cm^2^.

*Drug release test*

A glass Franz diffusion cell was used to evaluate the drug release profile. The receiver compartment was filled with 35 mL of phosphate buffer saline (pH 7.4) at 37 ºC. Two compartments were separated with a dialysis membrane with a cut-off size of 10 kDa. One milliliter of samples were withdrawn at the determined time intervals and suddenly replaced with 1 mL of PBS. The total concentration of Moxifloxacin in samples was determined by UV spectroscopy at the wavelength of 293 nm. 

*Scanning Electron Microscopy*

To describe the surface morphology of the wafers, sliced sections of each wafer were placed on double-sided adhesive carbon (bonded on stainless foundation). Then, the wafers were coated with a thin layer of gold under an argon atmosphere. The images were taken by scanning electron microscope (phenom proX, Netherlands) at an accelerating voltage of 5-15 kV.

*Differential scanning calorimetry*

A DSC instrument (Mettler Toledo DSC 232, Switzerland) was employed to determine the thermal behavior of wafers. For this, 3-5 mg of the wafer were placed into an aluminum pan and sealed using a crucible sealing press (Mettler Toledo, Switzerland). The process temperature was varied between 25 ºC to 500 ºC at a heating rate of 10 ºC/min under a nitrogen atmosphere (9).

*In-vitro antibacterial study*

Disc diffusion method was carried out to assess the antibacterial activity of wafers. *Pseudomonas aeruginosa* (*P. aeruginosa*) and *Staphylococcus aureus* (*S. aureus*) bacteria, mostly infectious strains in the wound, were used to determine the antimicrobial activity of wafers in comparison with Moxifloxacin disc (5 µg/disk). The bacterial counts were determined using half McFarland standard for each strain. The bacteria were spread into Mueller-Hinton agar and the discs were placed on. The discs of Moxifloxacin-loaded (DL) wafer, drug-free (DF) wafer, Moxifloxacin and drug free disc were used. The study was carried at 37 ºC incubation for 24 h. The experiment was carried out with three replicates and data was analyzed using ANOVA.

*In-vivo examination* of optimized wafer

Eight Healthy male BALB/c mice (25 g) were purchased from the animal Laboratory Resource Unit, Shahid Sadoughi University of medical science and health services. Animals were kept under temperature, humidity, and light-controlled environment and they have free access to the water and pellets. design of this study is adhered to Principles of Laboratory Animal Care and approved by Shahid Sadoughi University of medical science and health services Ethics committee.

Animals were divided into two groups of 4 mice. All the Mice were anesthetized with diethyl ether before shaving the surgical area. Two equal wounds with a diameter of 10 mm were created on the dorsal side of the mice body. The superior wound was treated without any treatment as the negative control and the other wound were treated with optimized DL or DF wafer. 

Because of the significant bioadhesion force of wafers, the wafers adhered to the wound during the experiment. Wound appearance and size were checked every day and photographs of each wound were captured every 2 days. Wounds were treated with DL or DF wafers were compared against the size, appearance, and histopathological evaluation.

At the end of the experiment, mice were euthanized using diethyl ether and specimens from wounded tissue were taken. Five micrometer sections from each specimen were cut using a microtome and stained with haematoxylin-eosin for pathologic evaluation of healed wounds.

## Results

Aimed to develop a delivery system with the modified release, various formulation of bilayer wafer was designed and examined. The optimization of variables was carried out after modeling with a Box-Behnken design.

*Scanning Electron Microscopy (SEM)*

**Figure 2:**
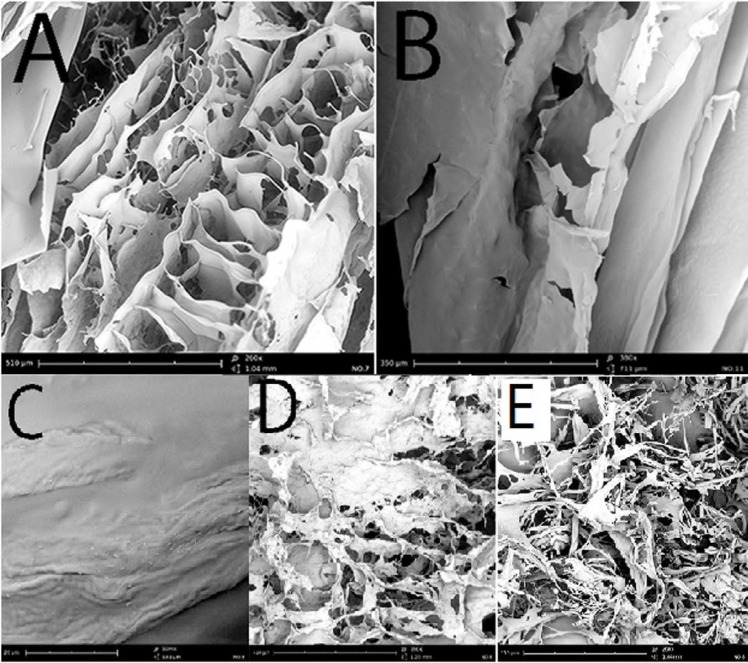
Scanning electron micrograph of (A) MOX + MC wafer SEM imaging (260x magnification), (B) MOX + HPMC wafer imaging (180x magnification), (C) Moxifloxacin crystals on the surface of PVP wafer, (D) MOX + gelatin wafer imaging (260x magnification), (E) MOX + PVP wafer SEM imaging (260x magnification)

The SEM imaging revealed the porous structures with interconnections in the microstructure of Moxifloxacin wafers. Whether HPMC or MC wafers demonstrated the sheet-shaped composition with interwoven fiber structure (Figures 2A-2B). Whilst, PVP and gelatin wafers exhibited more porous structure (Figures 2D-2E). Moxifloxacin crystals were another noticeable observation in the SEM pictures. Figure 2C shows Moxifloxacin crystals on the surface of MOX + PVP wafers. It seems Moxifloxacin molecule showed lower affinity to incorporate in the PVP matrix and deposited on the surface.

*Differential scanning calorimetry*

**Figure 3:**
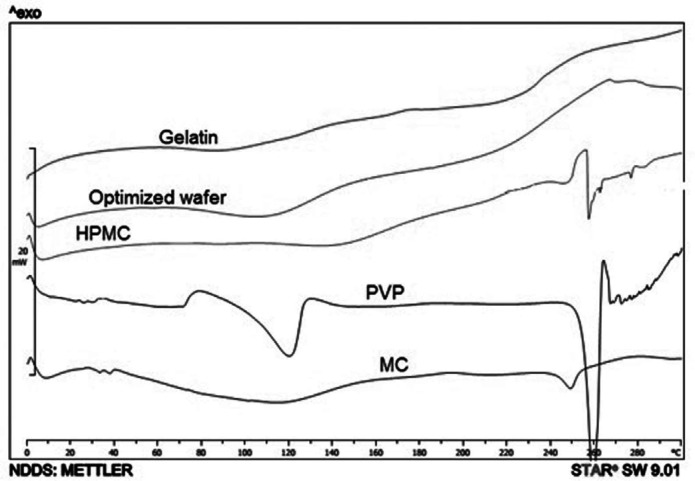
Thermograms of MOX + gelatin, MOX + HPMC, MOX + PVP and MOX + MC wafers. The sharp endothermic peak of MOX + PVP at 250 ºC is related to Moxifloxacin crystals

The thermograms of Moxifloxacin wafers are shown in Figure 3, MOX + PVP and MOX + HPMC wafers exhibited an endothermic peak at 258 ºC. This is related to the Moxifloxacin crystals. Moxifloxacin has different crystal habits and amorphous structures. For instance, alpha-1 Moxifloxacin with an endothermic peak at 250 ºC, and alpha-2 Moxifloxacin exhibits an endothermic peak at 253 ºC (10). Also, Moxifloxacin exhibited endothermic peaks at 213, 238, and 257 ºC at DSC thermogram in different studies (11-13). The different solid-state of drugs can influence the drug release profile from a polymeric matrix. For instance, entrapped drug in the polymeric matrix has a relatively slower drug release than a drug in crystalline form (14).

*Swelling behavior of wafers*

**Figure 4.**
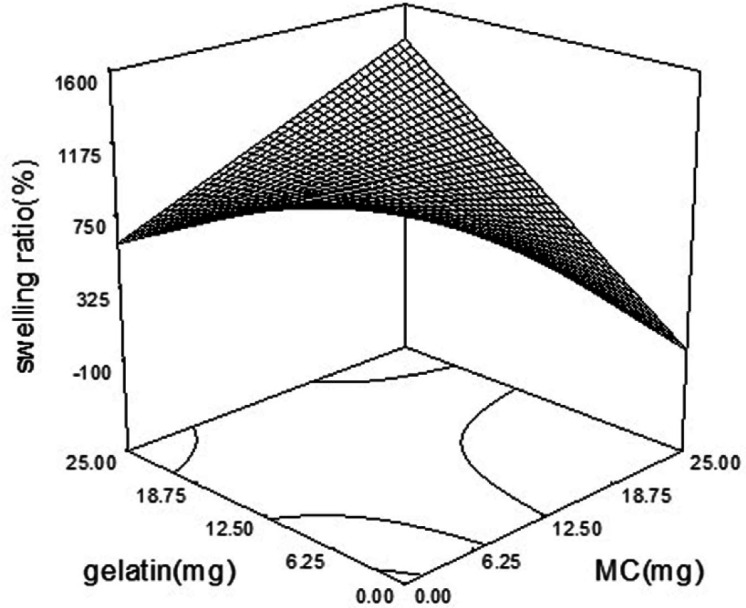
Interaction of gelatin and MC and its effect on swelling index. The highest swelling ratio is achievable when MC and gelatin are in their lowest concentration

The swelling ratio in various formulations was ranged from 230% to 1886%. A modified quadratic model (*p*-value = 0.0002) was fitted on swelling data. As is shown in Table 4, MC and gelatin with the coefficient of + 5 and *p*-value of 0.0005 and 0.0729, were the main factors affecting the swelling index of the wafers. Also, there was an additive effect between MC and gelatin. The simultaneous increase in the amount of MC and gelatin in the wafer boosted the swelling ratio (Figure 4). The incorporation of PVP as a highly water-soluble polymer as a pore-making agent (15) didn’t have a significant effect on the swelling ratio (*p*-value of 0.1829). But PVP and PG interaction showed a negative effect on swelling ratio by induction of wafer disintegration (16). MC is an amphiphilic polymer with hydroxyl groups that make it prone to hydrogen binding (17). Hydrophilic groups such as NH and OH increase the possibility of hydrophilic interaction between polymers and water, which is responsible for an increased swelling index of wafers containing MC and gelatin. 

Swelling ratio = + 1.084E + 006 + 2.171E + 005 × A - 3.475E + 005 × B + 4.808E + 005 × C + 3.358E + 005 × D + 2.326E + 005 × A × B - 2.050E + 005 × B × C - 1.510E + 005 × C × D + 1.988E + 005 × C2 + 1.327E + 005 × D2 

*Bioadhesion force of wafers*

Bioadhesion is one of the most important properties of bioadhesive systems (18). Wafers should be enough bioadhesive to provide a long time resistance on the wound area. The bioadhesive force of wafers was varied between 1.1 and 2.1 N/cm^2^ in all formulations.

A modified 2FI model (*p*-value = 0.0079) was fitted on wafers bioadhesion with following equation:

Bioadhesion = + 373.81 + 138.05 × A + 66.43 × B + 194.44 × C + 0.73 × D - 194.27 × A × B + 114.72 × A × C + 89.67 × B × C

**Figure 5:**
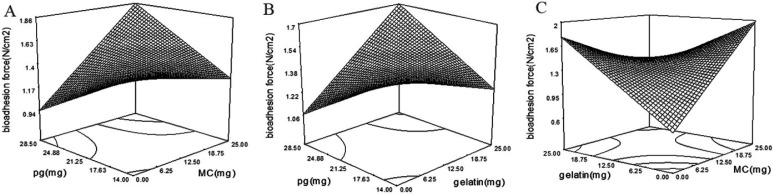
(A) Interactions of PG and MC, (B) PG and gelatin, and (C) gelatin and MC, and their effect on bioadhesion force of wafers. Addition of PG to wafers contained MC or gelatin increase the bioadhesion force

There are different mechanisms explaining bioadhesion. In summary, bioadhesion strength is a result of different factors such as electrical interactions, hydrophilic interactions, and interference of polymer chains with mucin (18). By increasing the polymer hydrophilicity, the bioadhesive strength will increase subsequently. Also, polymer chain flexibility influences bioadhesive strength (18). Existing of plasticizers in the matrix enhances the flexibility of the films by disrupting the intermolecular forces between the polymer chains (19). It is considered that High polymer flexibility is favorable for bioadhesion (20). The addition of plasticizer to a system by changing the surface properties of polymers plays a crucial role in the bioadhesivity of the system (21). The same results were observed with Eudragit tablets and HPC films. Which higher hydrogen bonding was observed after plasticization (22, 23). Incorporation of PG in the wafer matrix as a plasticizer enhanced bioadhesivity with the *p*-value of 0.0058 and coefficient of + 194.44 (Table 4). Also, bioadhesivity of wafers affected by MC and gelatin interaction with a *p*-value of 0.0004 and coefficient of -194.27. According to Figure 5C by increasing the amounts of MC or gelatin in formulation, the bioadhesivity will increase. But, by concurrent increasing in MC and gelatin the bioadhesivity will decrease. The lowest bioadhesive strength is achievable at lowest concentration of MC and gelatin. HPMC, MC and gelatin are known as bioadhesive polymers (24). By increasing the concentration of MC and gelatin in wafers, the bioadhesion of wafers will increase. But, by simultaneous increase of MC and gelatin in formulation, the bioadhesivity decreased. However, MC and gelatin positively affected the wafer adhesion, but their interaction is negative (coefficient equal to -194.27). Polymer coiling which happens at a higher concentration of polymers in the polymeric matrix can be responsible for this interaction (18). At higher polymer concentrations, the coiled polymer chains lose their flexibility (25) which is responsible for reduced bioadhesion of wafers at higher concentrations of MC and gelatin (26).

The significant interaction of PG with MC and gelatin with the coefficient of 114.72 and 89.67 respectively, increased the bioadhesivity. According to Figure 5A and B, by an increase in the concentration of PG, the bioadhesion of MC and gelatin increases. Viscosity is one of the most important factors affecting bioadhesion. The plasticized polymers expose lower viscosity than the unplasticized polymers (27). Also, Plasticizers reduce the intra-molecular interactions which are responsible for increased bioadhesion force of plasticized polymers (28).

*Drug release profile of wafers*

The biphasic drug release profile was predictable because of the bilayer structure of wafers. For this, the wafer drug release was evaluated with the time lasted to 40% and 90% of loaded drug release from the wafer (T_40_ and T_90_). Also, the drug release behavior was modeled by the fitting of the released drug against time and evaluating the maximum R^2^.

The drug release pattern from the wafers was modeled with Higuchi, zero-order and first-order model which the R^2^ of the Higuchi model was found to be higher than Zero- and first-order (Table 5). 

When the solubility of a solute is lower than its concentration, the Higuchi model explains the drug release profile. In this model, by exhausting of matrix surface from the drug at sink conditions, the next layer drug starts to dissolve into a solvent (29). Drug release profiles from HPMC matrixes followed the Higuchi model as reported previously (30-32). As is shown, a modified quadratic model with a *p*-value of 0.0001 was fitted on drug release rate constant with the following equation:

 Releas Rate = -1276.75 + 458.91 × A + 926.91 × B - 1314.25 × C - 79.31 × D + 486.96 × A × B + 351.46 × C × D + 249.52 × B2 - 416.02 × C2 - 186.65 × D2

**Figure 6:**
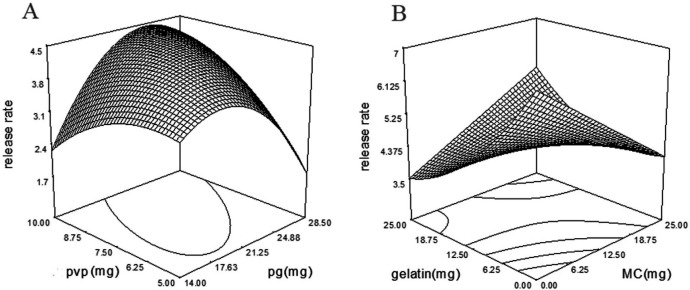
(A) Interaction of PVP and PG and (B) MC and gelatin, and their effect on drug release rate. Simultaneous increase in amounts of PVP and PG in the wafer formulation increase the drug release rate

According to Table 4, incorporation of MC (*p*-value = 0.0002, coefficient = 458.9) and gelatin (*p*-value = 0.0001, coefficient = 926.9) facilitated drug release from wafers. But, simultaneous increase of MC and gelatin in the formulation sustained drug release (Figure 6B). PG with the *p*-value of 0.0260 and coefficient of -1314.25 reduced the drug release rate, but, PVP and PG interaction with the *p*-value of 0.0014 and coefficient of 351.46 increased drug release rate (Figure 6A).

A modified quadratic model with *p*-value of 0.0004 was fitted on T_40_ with following equation:

T_40_ = + 20706.64 + 16900 × A-46770.28 × B-9466.33 × C–35180.54 × D-27797.80 × A × B + 26903.33 × A × C-16622.43 × A2-11005.54 × B2

Fast releasing of moxifloxacin from wafers is important to have a loading dose at the wound. According to Table 4, gelatin was the main factor affecting T_40_. Gelatin with the *p*-value of 0.0017 decreased the T_40_ time. As is shown in the DSC thermogram of gelatin + MOX wafer (Figure 3), the Moxifloxacin crystals are evident in wafers as well as PVP + MOX wafers. It is predictable that MOX crystals on the gelatin and PVP surface are capable to freely release from the matrix. Also, the interaction of MC and gelatin with a *p*-value of 0.0053 subtracts the T_40_ time which is in accordance with the release rate.

The remained drug will release from the second layer at a slower rate. A modified quadratic model with *p*-value of 0.0004 was fitted on T_90_ data and followed below equation:

T_90 _= -13027.14 + 1629.82 × A - 1.567E + 005 × B + 60043.56 × C - 18640.42 × D - 83171.38 × A × C + 46398.08 × A × D - 56630.84 × B × C - 69955.10 × C × D + 53284.45 × A^2^ + 70050.92 × C^2^ + 23276.52 × D^2^

As shown in Table 4, gelatin with the *p*-value of 0.0295 and coefficient of -1.567E + 005 was the main factor affecting T_90_; increasing in gelatin in wafer formulation decreased the T_90_. Similarly, gelatin reduced T_40_ and increased the drug release rate as mentioned above. Surprisingly, the data revealed that by the interaction of propylene glycol and PVP in the formulations; the T_90 _decreased which is comparable with the same interaction on drug release rate. Propylene glycol is known as a plasticizer (33) and co-solvent which increases drugs solubility (34). Moxifloxacin hydrochloride, a polarized antibiotic, is a sparingly soluble drug (19.6 mg/mL) (34). PG enhances the drug solubility, but after reaching a critical concentration decreases the flux efficiency. Which is responsible for the inhibitory effect of PG on the drug release rate (35).

*Optimization*

Aimed to get the best formulation with optimized properties the predicted optimized formulation was obtained by the optimum value of each excipient. The optimum formulation and predicted values of each component are shown in Table 6. 

*In-vitro anti-bacterial efficacy of optimized wafer*

An antimicrobial efficacy test was applied to evaluate the inhibitory properties of optimized Moxifloxacin-loaded wafers and Moxifloxacin disc against most infectious pathogens in wounds. Results revealed that Moxifloxacin wafers have equal efficacy in comparison with Moxifloxacin discs (*p*-value < 0.05). Suitable design of bilayer wafer with biphasic order of release provided the effective loading dose and resulted in equal average Zone of inhibition (ZOI) of Moxifloxacin against *p. aeruginosa* and *s. aureus* with Moxifloxacin discs that are summarized in Table 7.

*In-vivo wound healing experiment*

**Figure 7:**
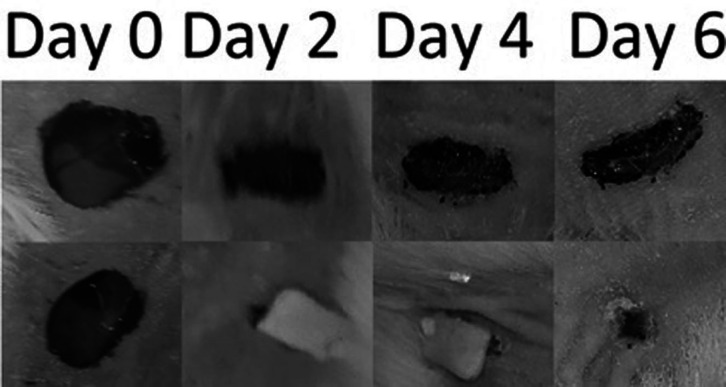
Size and appearance of DL wafer treated wounds during the experiment. Continues shrinkage of wafer in wound fluids is evident. By shrinkage of wafer in the wound exudates, released moxifloxacin from wafer inhibits the wound infection and promote wound healing

To evaluate the efficacy of the optimized wafer *in-vivo* a wound-healing experiment was carried out using an animal model. After the application of the optimized wafers on the wounds, the wafers adhered to the wound tissue immediately due to their bioadhesivity. Also, all wafers were resisted on the wounds at least for four days after application. As shown in Figure 7 wafers are capable to adhere to the wound up to the end of the experiment and wound healing duration. Also as depicted in Figure 7 the wafer shrunk during the time that the wafer was adhered to the wound. Wafers started to put off the wound’s surface just after the wound closure. The wounds were treated with MOX-loaded wafers healed without any sign of infection. Data showed that the wounds were treated with Moxifloxacin-loaded wafers, healed 6 days faster than their control wounds. Also, the wounds treated with drug-free wafers healed 3 days faster than their control wounds.

**Figure 8:**
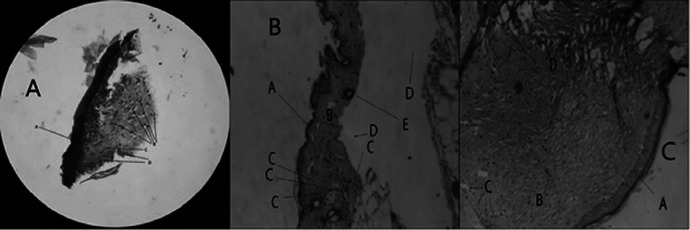
(A) Microscopic view of DL wafer treated wound (H&E staining, 100x magnification). The wounds were treated with DL wafers express more fibroblasts and less inflammation. (A) epidermis, (B) fibroblast cells, (C) collagen fibers, (D) inflammatory cells. (B) Microscopic view of wound without receiving any treatment. Mature collagens and lake of fibroblasts are evident (H&E staining, 100x magnification). (A) epidermis, (B) dermis, (C) inflammatory cells, (D) artifact, (E) hair follicle. (C) Microscopic view of drug free treated wound (H&E staining, 100x magnification). (A) epidermis, (B) fibroblast cells, (C) inflammatory cells, (D) collagen fibers

The histologic study was carried to determine and compare wound healing properties of DL and DF optimized wafers. Pathological observations proved the effective healing properties of wafers after treatment. As shown in the Figure 8 the epithelium was completely recovered in the wounds were treated with a DL wafer. Also, well-organized fibroblasts, lack of inflammation, mature fibrous tissue, and absence of pathologic abnormalities in the wound treated with DL wafer are supporting this idea. The wounds were treated with DF wafers were healed as well as DL wafers, but inflammatory cells appear in higher numbers. On the other hand, the presence of mature collagen, the lake of fibroblasts and heavy inflammation are evident in the control wounds. 

Fibroblast cells play a crucial role in the wound healing process such as the promotion of the formation of a new extracellular matrix (ECM). Also, fibroblasts are necessary for the contraction of wounds and the production of fibrin clots (36). One of the most challenging strategies in wound healing is providing an ideal microenvironment for optimal cell migration (37). Tissue engineering studies have relied on the creation of three-dimensional extracellular matrices (ECM) to guide cell adhesion, growth and differentiation to form a functional tissue (37). Artificial ECMs can prevent wound environment from infection and provide appropriate conditions for fibroblast migration (38). The ECM-based system showed an obvious effect on the wound healing parameters when loaded with antibiotics and growth factors (39). Biodegradable hydrophilic materials in hydrogels may promote cell adhesion, tissue regeneration and wound healing. A form of biodegradable scaffold formulations showed effective wound healing outcomes (40). Also, to prevent infection in the wound, an antibacterial agent would be helpful (41). Moxifloxacin as a fourth-generation fluoroquinolone is a broad-spectrum antibiotic that is active against both Gram-positive and Gram-negative bacteria. On the other hand, Moxifloxacin exhibited wound healing properties in a recent study (42) The combination of three-dimensional porous ECM of biodegradable polymers and Moxifloxacin as an antibiotic promoted wound healing in this study.

**Table 1:** Dependent and independent variables introduced to Design Expert software

**Independent variable**	**dependent variable**
Gelatin conc.	Bioadhesion force
Propylene glycol (PG) conc.	Release rate
Methylcellulose (MC) conc.	Swelling ratio
Polyvinylpyrrolidone (PVP) conc.	T_40_
	T_90_

**Table 2:** The high and low levels of independent variables introduced to Design Expert software

	**Independent variables**	**-1 Level**	**+ 1 Level**
A	MC	0	25
B	Gelatin	0	25
C	PG	14	28
D	PVP	5	10

**Table 3:** Various wafer formulation acquired from Design Expert software and Box-behnken design

**Runs**	**HPMC**	**MC**	**Gelatin**	**PVP**	**PG**	**MOX**
Run 1	50	25	12.5	10	24	5
Run 2	50	12.5	0	10	18	5
Run 3	50	0	12.5	7.5	14	5
Run 4	50	0	12.5	10	18	5
Run 5	50	12.5	25	10	24	5
Run 6	50	12.5	12.5	5	16	5
Run 7	50	12.5	25	5	23	5
Run 8	50	25	12.5	5	23	5
Run 9	50	12.5	25	7.5	29	5
Run 10	50	12.5	12.5	7.5	21	5
Run 11	50	12.5	12.5	7.5	21	5
Run 12	50	12.5	12.5	10	26	5
Run 13	50	0	12.5	5	17	5
Run 14	50	0	0	7.5	14	5
Run 15	50	25	12.5	7.5	29	5
Run 16	50	12.5	0	7.5	14	5
Run 17	50	12.5	12.5	7.5	21	5
Run 18	50	12.5	0	5	17	5
Run 19	50	25	0	7.5	21	5
Run 20	50	0	25	7.5	21	5
Run 21	50	12.5	0	7.5	21	5
Run 22	50	0	12.5	7.5	21	5
Run 23	50	25	25	7.5	27	5
Run 24	50	12.5	12.5	10	17	5
Run 25	50	12.5	12.5	5	24	5
Run 26	50	25	12.5	7.5	19	5
Run 27	50	12.5	25	7.5	19	5
Run 28	50	12.5	12.5	7.5	21	5
Run 29	50	12.5	12.5	7.5	21	5

**Table 4:** Results of ANOVA test for swelling ratio, Bioadhesion force, T40, T90 and drug release rate

**T**_90_	**T**_40_	**Release rate**	**bioadhesion**	**swelling**	
*p*-value	*p*-value	*p*-value	*p*-value	*p*-value	
0.009	0.0004	<0.0001	0.0079	0.0002	model
0.3956	0.9835	0.0002	0.2388	0.0005	A-MC
0.0017	0.0295	<0.0001	0.5658	0.0729	B-Gelatin
0.6491	0.6344	0.0260	0.0058	0.1891	C-PG
0.0740	0.7908	0.8443	0.5061	0.1829	D-PVP
0.0032	-	0.0001	0.0004	0.0004	AB
0.0129	0.019	-	0.0198	-	AC
-	0.009	-	-	-	AD
-	0.0282	-	0.062	0.0078	BC
-	-	-	-		BD
0.0785	0.0019	0.0014	-	0.0172	CD
0.0152	0.0014	-	-	-	A^2^
0.0264	-	0.0008	-	-	B^2^
-	0.024	0.0003	-	0.0075	C^2^
-	0.0423	0.0063		0.0018	D^2^
0.9539	0.0665	0.9976	0.4661	0.4568	Lack of fit

**Table 5:** R^2^ values of different formulations after plotting released drug against time

**runs**	**Higuchi**	**first order**	**Zero order**	
1	0.996	0.978	0.973	
2	0.99	0.735	0.974	
3	0.996	0.706	0.973	
4	0.996	0.719	0.973	
5	0.991	0.784	0.982	
6	0.997	0.769	0.98	
7	0.99	0.749	0.749	
8	0.995	0.479	0.899	
9	0.995	0.785	0.983	
10	0.973	0.775	0.956	
11	0.977	0.829	0.991	
12	0.996	0.769	0.97	
13	0.995	0.691	0.971	
14	0.996	0.779	0.98	
15	0.989	0.834	0.998	
16	0.992	0.486	0.915	
17	0.957	0.655	0.984	
18	0.995	0.646	0.98	
19	0.964	0.724	**0.95**	
20	0.987	0.747	0.993	
21	0.992	0.687	0.968	
22	0.998	0.816	0.98	
23	0.99	0.769	0.97	
24	0.986	0.847	0.99	
25	0.996	0.953	0.994	
26	0.947	0.692	0.968	
27	0.988	0.771	0.986	
28	0.995	0.688	0.978	
29	0.994	0.593	0.985	
				
			

**Table 6:** Predicted values of optimized formulations by DX7 software

**HPMC**	**MC**	**gelatin**	**PG**	**PVP**	**Bioadhesivity**	**Swelling ratio**	**T**_90_	**T**_40_	**Desirability**
50 mg	25 mg	2 mg	8.05 mg	10 mg	1.87N/ Cm^2^	1840.22%	1409.4 min	89.19 min	0.851

**Table 7:** Average ZOI area (mm) of Moxifloxacin disc (three replications) in comparison with Moxifloxacin wafer in *P. aeruginosa* and *S. aureus*

	***P. aeruginosa***	***S. aureus***
Moxifloxacin disc	9.72	11.27
Moxifloxacin wafer	9.49	11.20

## Conclusion

The optimum topical bilayer wafer of moxifloxacin was developed with the aid of soluble polymer including hydroxypropyl methylcellulose, gelatin, methyl cellulose, Polyvinylpyrrolidone and propylene glycol as a plasticizer. Inclusion of gelatin and methyl cellulose by composing the wafer backbone help to form a swellable, bioadhesive layer with controlling the drug release. The addition of PG and PVP to the porous layer made it suitable for the fast release of drugs. Also, the incorporation of PG in formulation increased the wafer bioadhesive force. Altogether, the application of optimized formulation leads to healing of wound 6 days faster without any sign of infection.
